# Sucrose phosphate synthase (SPS), sucrose synthase (SUS) and their products in the leaves of *Miscanthus* × *giganteus* and *Zea mays* at low temperature

**DOI:** 10.1007/s00425-020-03421-2

**Published:** 2020-07-16

**Authors:** Anna Bilska-Kos, Jennifer Mytych, Szymon Suski, Justyna Magoń, Piotr Ochodzki, Jacek Zebrowski

**Affiliations:** 1 Department of Plant Biochemistry and Physiology, Plant Breeding and Acclimatization Institute, National Research Institute, Radzików, 05-870 Błonie, Poland; 2 Department of Plant Physiology and Ecology, Institute of Biology and Biotechnology, University of Rzeszow, Aleja Rejtana 16c, 35-959 Rzeszow, Poland; 3 Department of Animal Physiology and Reproduction, Institute of Biology and Biotechnology, University of Rzeszow, Werynia 2, 36-100 Kolbuszowa, Poland; 4 Laboratory of Electron Microscopy, Nencki Institute of Experimental Biology of Polish Academy of Sciences, 3 Pasteur Str, 02-093 Warsaw, Poland; 5 Department of Plant Pathology, Plant Breeding and Acclimatization Institute, National Research Institute, Radzików, 05-870 Błonie, Poland

**Keywords:** Cellulose, Immunolocalization, Starch grains, Sucrose, Sugar metabolism

## Abstract

**Main conclusion:**

The changes in the expression of key sugar metabolism enzymes (SPS and SUS), sucrose content and arrangement of chloroplast starch may play a significant role in the cold response in *M. giganteus* and maize plants.

**Abstract:**

To understand the mechanism of the chilling-response of two closely-related C_4_ plants, we investigated the changes in the expression of sucrose phosphate synthase (SPS) and sucrose synthase (SUS) as well as changes in their potential products: sucrose, cellulose and starch in the leaves of *Miscanthus* × *giganteus* and *Zea mays*. Low temperature (12–14 °C) increased SPS content in *Miscanthus* (MG) and chilling-sensitive maize line (Zm-S), but not in chilling-tolerant one (Zm-T). In Zm-S line, chilling also caused the higher intensity of labelling of SPS in the cytoplasm of mesophyll cells, as demonstrated by electron microscopy. SUS labelling was also increased by cold stress only in MG plants what was observed in the secondary wall between mesophyll and bundle sheath cells, as well as in the vacuoles of companion cells. Cold led to a marked increase in total starch grain area in the chloroplasts of Zm-S line. In turn, Fourier transform infrared spectroscopy (FTIR) showed a slight shift in the cellulose band position, which may indicate the formation of more compact cellulose arrangement in Zm-T maize line. In conclusion, this work presents new findings supporting diversified cold-response, not only between two C_4_ plant species but also within one species of maize.

## Introduction

Sucrose phosphate synthase (SPS, EC.2.4.1.14) and sucrose synthase (SUS, EC.2.4.1.13) are key enzymes in the sugar metabolic pathways in the plants. SPS catalyzes the conversion of fructose-6-phosphate and uridine diphosphate-glucose (UDP-glucose) into sucrose-6-phosphate which is a substrate in the synthesis of sucrose (Winter and Huber 2000; Chen et al. 2005). SUS catalyzes the conversion of sucrose and nucleoside diphosphate into the corresponding nucleoside diphosphate-glucose and fructose, and provides substrates for cellulose and starch biosynthesis: UDP-glucose and adenosine diphosphate-glucose (ADP-glucose) (Amor et al. 1995; Haigler et al. 2001; Baroja-Fernández et al. 2009).

SPS and SUS exist in many isoforms which may play various functional roles and be specialized in different tissues and stages of development. In addition, the activity and the localization of these enzymes can be controlled by reversible protein phosphorylation through the calcium-dependent kinases (CPDKs) and regulation mechanisms in individual species are varied (Huber and Huber 1996; Toroser and Huber 1997; Winter et al. 1997; Foyer et al. 1998; Duncan et al. 2006; Fedosejevs et al. 2014; Almadanim et al. 2017).

The determination of the functional roles of these isoforms is an ongoing area of research. Particularly, knowledge about the relationship of localization of individual forms of these enzymes and their role in plant responses to various stresses is highly desirable. It has been demonstrated for instance, that several maize SPS sequences were most strongly expressed in the leaves and less intensively in pollen and kernel, what was related to the reaction to different abiotic factors (Lutfiyya et al. 2007). In turn, in the columella cells of *Arabidopsis thaliana* roots, SPS isoforms support sucrose synthesis and stimulate starch synthesis in this tissue and respond differentially to the osmotic stress (Solís-Guzmán et al. 2017). In the source leaves of *Nicotiana tabacum*, the differential expression of three SPS isoforms revealed their involvement in the starch mobilization during the dark period (Chen et al. 2005). Similarly, four SPS isoforms localized in source leaves of *Arabidopsis thaliana* plants can play a role in dark respiration via enhancement of starch turnover (Bahaji et al. 2015).

SUS isoforms have been found in the different subcellular localizations: in vacuoles (Etxeberria and Gonzalez 2003), in plastids (Núñez et al. 2008), in mitochondria (Subbaiah et al. 2006), in the cytosol (Baroja-Fernández et al. 2009) as soluble enzyme and also in a membrane-bound form (Winter et al. 1997), and in the different areas of the cell wall (Salnikov et al. 2003; Persia et al. 2008). The different localization of individual SUS isoforms can promote the membrane-associated or cytosolic sucrose degradation which may result in the further type of carbon utilization path, i.e., cellulose or starch synthesis what can be additionally regulated by various stress factors. For instance, SUS localized in the plasma membrane in the developing cotton fibers can utilize carbon directly from sucrose to cellulose and/or callose synthases (Amor et al. 1995). In maize, the specificity of the function of individual SUS isoforms in both cytoplasmic and membrane-associated sucrose degradation was emphasized (Duncan et al. 2006). Also, in hexaploid wheat, the two types of SUS genes were differentially expressed in the endosperm, leaves and roots in response to anaerobiosis, light conditions and cold shock (Maraña et al. 1990).

The effect of low temperature on SPS and SUS activity has been studied in various approaches, mainly in C_3_ plants, by determination of changes in the expression of the genes and/or proteins as well as by analysis of sucrose and starch content. In general, during cold-response of plants, an increase in sucrose level is widely observed (reviewed by Sage and Kubien 2007). In the leaves of cold-treated spring and winter wheat, an increase in sucrose amount was noted, but these plants were not capable of adjusting the SPS and SUS activity to stress conditions (Savitch et al. 2000). Similarly, low temperature caused disorders in the development of wheat spikelets and florets via sucrose over-accumulation as well as changes in enzymes activity and gene expression associated with sucrose metabolism (Zhang et al. 2019). In the chilling-sensitive accession of *Arabidopsis* plants, sucrose synthesis is indicated as a metabolic ‘bottleneck’ in response to cold conditions (Nägele et al. 2012). Guy et al. (1992) suggested that the increase in SPS activity and sucrose level in spinach leaves at low temperature is one of the adaptive mechanism elements manifested by the accumulation of compounds with cryoprotectant properties.

Following the above literature data, the presented studies on the expression of key plant sugar metabolism enzymes, their (sub) cellular localization and their potential products in cold-stressed C_4_ plants seem to be an important and original research approach. Our earlier studies support this approach, where diverse physiological, anatomical and biochemical responses to cold of *Miscanthus* and maize plants were found, including the changes in the composition of the neutral cell wall polysaccharides (Bilska-Kos et al. 2018). The results may suggest that the activity of the key enzymes in sugar metabolism can play a crucial role in C_4_ plants response to chilling. The aim of the present work was to verify the hypothesis that low temperature affects the abundance and/or localization of SPS and SUS1, as well as the organization of their potential products—sucrose, cellulose and starch. For this purpose, Western blotting and electron microscopic immunolocalization of these enzymes were performed. We analyzed the sucrose level using gas chromatography, as well as the cellulose organization by infrared spectroscopy (FTIR) in the leaf cell wall of the tested plant species. We also discuss the possible importance of the changes in the arrangement of starch grains in the chloroplasts in the context of the response to cold in the tested plants. Our work will provide valuable, previously unknown information about cold tolerance/sensitivity mechanism of C_4_ plant.

## Materials and methods

### Plant material and growth conditions

The leaves of *Miscanthus* × *giganteus* (MG) and two maize lines (*Zea mays* L.): chilling-tolerant S68911 (Zm-T) and chilling-sensitive B73 (Zm-S) were used as an experimental material. The material was obtained from the following sources: *Miscanthus* rhizomes from the private plantation (Majdan Sieniawski, Poland), S68911 maize seeds from Plant Breeding Smolice Co., Ltd. (Smolice, Poland) and B73 maize seeds from USDA, ARS, Iowa State University (Ames, Iowa, USA). Both tested maize lines: S68911 and B73 are inbred lines belonging to *Z. mays* spp. *indentata* (dent type) representing Stiff Stalk Synthetic/Iodent pool and are characterized in the transcriptomic studies for their cold tolerance/sensitivity (Revilla et al. 2016; Sobkowiak et al. 2016). Plants were grown in a 5-L pots with a solid substrate in controlled conditions, in a growth chamber with parameters: 14/10 h light/darkness, irradiance 350 µmol quanta m^−2^ s^−1^ at 24/22 °C (day/night) temperature. At the stage of the third fully developed leaf (ligular region present), the plants were transferred to low temperature 14/12 °C (day/night) for 28 h. Chilling treatment was started at the beginning of the light period (chilling treatment period: 14 h/light, 10 h/dark, 4 h/light) and the control samples were taken 4 h after the light had been switched on. In each repetition of three independent experiments at least 6 plants per variant/per analysis type were used.

### Western-blot analyses

The total protein from *M. giganteus* and maize leaves was extracted with 50 mM Tris–HCl pH 7.5, 100 mM NaCl, 0.2% Triton-X, 0.05% β-mercaptoetanol, 6 M urea, 2 mM PMSF and denatured with 5 × Laemmli Sample Buffer at 70 °C for 5 min. Next, 30 µg of protein samples were separated on 10% SDS-PAGE and electroblotted for 100 min to PVDF using tank transfer. Blots were blocked with 5% milk in TBS-T for 30 min at room temperature (RT) with agitation. Blots were incubated with primary antibodies: anti-actin (A0480, Sigma-Aldrich), anti-SPS global (AS03 035A, Agrisera, Vännäs, Sweden) or anti-SUS1 (AS15 2830, Agrisera) at a dilution of 1:1000 overnight at 4 °C (antibodies diluted in 1% BSA in TBS-T). The antibody solution was decanted and the blots were washed four times for 5 min in TBS-T at RT with agitation. Blots were incubated for 1 h at RT with agitation with secondary antibodies diluted in 1% BSA in TBS-T: anti-mouse IgG horseradish peroxidase conjugated (A9044, Sigma Aldrich) 1:40,000 (actin), anti-rabbit IgG horseradish peroxidase conjugated (AS09 602, Agrisera)—1:10,000 (SPS) and 1:40,000 (SUS1). The blots were washed as above and developed for 5 min with Clarity Max Western ECL Substrate (Bio-Rad Laboratories, Hercules, CA, USA). Exposure time was 30 s (for SPS) or 60 s (for SUS1).

### The preparation of material for electron microscopy

Leaf samples were fixed in 4% paraformaldehyde with 0.5% glutaraldehyde in 0.1 M PB (phosphate buffer), pH 7.3 at 4 °C for 4 h. After washing (PB) and dehydration (ethanol, 10–100%) the material was embedded in LR White resin (Sigma-Aldrich) and polymerized for 168 h at 37 °C. Ultrathin  sections (80 nm) mounted on nickel grids were obtained using a Leica Ultracut UTC ultramicrotome.

### Immunogold localization

The immunolocalization of SPS and SUS was performed accordingly to the procedure previously described by Bilska-Kos et al. (2016b). Briefly, in the first step, the unspecific epitopes were blocked with 4% bovine albumin (BSA) in phosphate-buffered saline (PBS, 0.01 M, pH 7.3). After washing in the washing mixture (1% BSA/PBS), samples were incubated for 2 h with primary antibodies, for SPS: anti-SPS global (AS03 035A, Agrisera) or for SUS: anti-SUS1 (AS15 2830, Agrisera) in the dilution: 1:100 (in PBS). For the negative control, the incubation with primary antibodies was omitted. After a series of washings—1% BSA (in PBS), PBS and water—material was incubated for 2 h with secondary antibodies conjugated to 10 nm gold particles (goat anti-rabbit, Sigma-Aldrich). After washing (water) samples were contrasted with uranyl acetate (2.5%) for 20 min and lead citrate (0.04%) for 15 min. The observations were performed using a transmission electron microscope (JEM 1400; Jeol Co., Tokyo, Japan) equipped with an 11-mpx MORADA G2 camera (EMSIS GmbH, Münster, Germany).

### Sucrose content

The concentration of sucrose was determined as trimethylsilyl ether by gas chromatography with flame ionisation detection (GC-FID) according to Knudsen and Li (1991) with minor modifications. Briefly, 10 mg of freeze-dried leaf material was extracted with 1 ml of 80% (v/v) ethanol and 100 µl of internal standard (phenyl β-D-glucose, 5 mg ml^−1^) at 60 °C for 60 min. with occasional shaking. After cooling and centrifugation, clear supernatant (500 µl) was transferred to 4-ml vials and evaporated to dryness in stream on nitrogen. Dry residues were re-dissolved and derivatized to trimethylsilyl ether with 75 µl of Sylon BTZ (Sigma-Aldrich). Next, after cooling, 1 ml of isooctane and 2.5 ml of deionised water were added and intensively shaken. After separation, the organic layer containing derivatized sucrose was transferred to 1.5 ml vials and analyzed with GC-FID. Chromatographic analysis of sucrose was conducted on SRI 8610C gas chromatograph (SRI Instruments, Torrance, CA, USA), fitted with flame ionization detector (GC-FID) and HTA200 autosampler (HTA S.r.l., Brescia, Italy). Solutes were separated on BGB-5MS capillary column (30 m, 0.25 mm ID, 0.25 µm) in temperature gradient (from 160 °C to 280 °C, 5 °C min^−1^). Data acquisition and integration were conducted with PeakSimple ver. 454 integration programme. For the quantitative analysis of sucrose content the calibration curve was constructed, linear in the range of concentrations 0.1–1.0 mg ml^−1^ (*r*^2^ = 0.999).

### Fourier transform infrared (FTIR) spectroscopy

For the analysis of cellulose organization in leaves of *M. giganteus* and maize plants, the cell wall was isolated from frieze-dried leaves and purified from starch. The mid-infrared spectra were collected using Fourier transform infrared spectrometer (FTIR) Nicolet iZ 10 module (Thermo Fisher Scientific) and the ATR accessory equipped with a diamond crystal. Interferograms (256) were gathered at the resolution of 4 cm^−1^ and co-added within the wavelength range between 4000 and 400 cm^−1^ using OMNIC (v. 8.1, Thermo Fisher Scientific) software. The spectra were normalised to the unit area (1800–900 cm^−1^) after the baseline correction and processed for analysis using the ChemoSpec (Hanson 2017) and the hyperSpecc (Beleites and Sergo 2018) packages in the R (R Core Team 2018).

### Starch grains organization

Based on the electron microscope images, the percentage of the starch granules area in the total area of chloroplast was determined using iTEM software (Olympus Soft Imaging Solution). The analysis was performed on the chloroplasts of Kranz mesophyll (KMS) and bundle sheath cells (BS). For the quantitative analysis of starch grains, at least thirty KMS and BS cells per experimental variant were considered.

### Statistical analysis

The analysis of significant differences between mean values was performed using two-way ANOVA followed by the pairwise multiple comparisons of means using ‘post-hoc’ Tukey’s Honestly Significant Difference (HSD) test. Both main effects: the genotype (three levels) and the treatment (two levels) as well as the genotype x treatment interactions were taken into account in the model. For analysis of cellulose peak position in FTIR studies, additionally 95% Confidence Intervals (CIs) were calculated. The differences were considered significant for *P* value less than 0.05. The analysis was performed using R programming (R Core Team 2018).

## Results

### Western-blot analysis

**Fig. 1:**
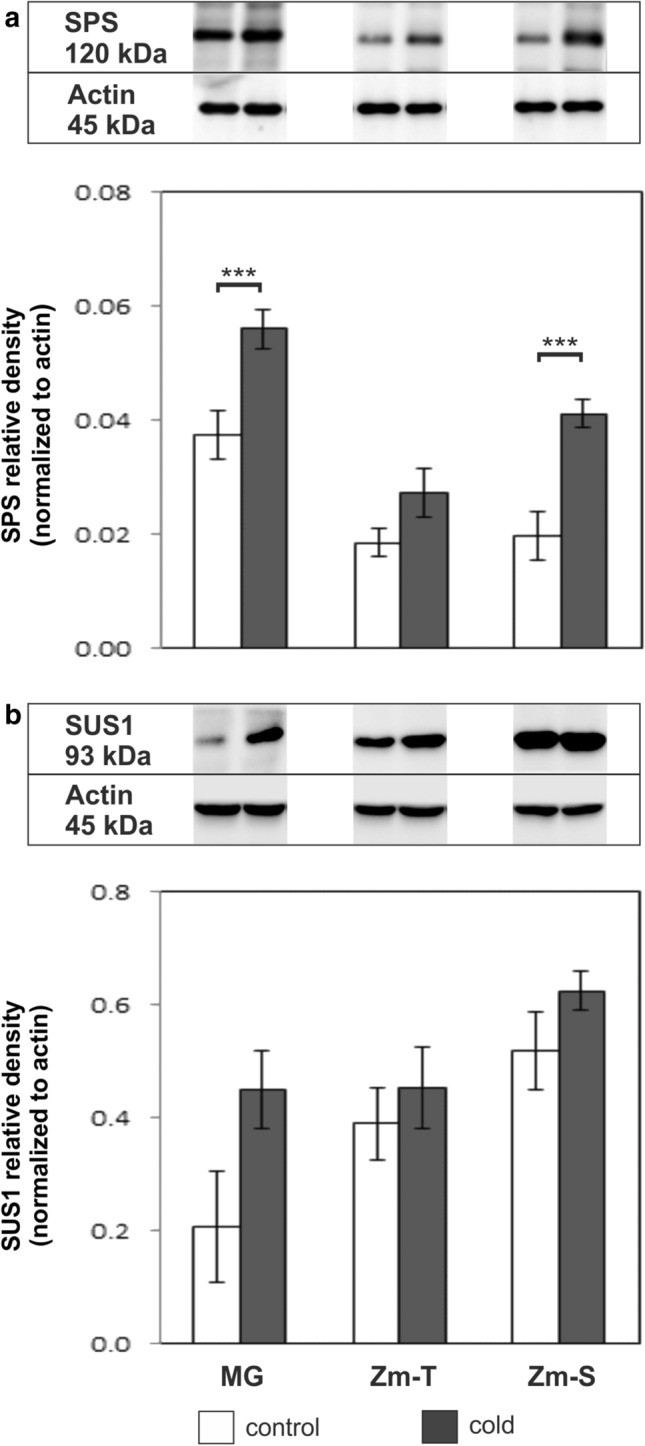
The relative expression of **a** sucrose phosphate synthase (SPS) and **b** sucrose synthase 1 (SUS1) in leaves of the control (white bars) and chilled (grey bars) plants of *Miscanthus* × *giganteus* (MG), chilling-tolerant (Zm-T) and chilling-sensitive maize line (Zm-S). Representative immunoblots are reported. The normalization was performed relative to actin. **a** For SPS, there is a significant effect of genotype [ANOVA, *F *(2;12) = 68.73; *P* < 0.0001], treatment [ANOVA, *F *(1;12) = 90.83; *P* < 0.0001] and of the interaction of genotype: treatment [ANOVA, *F *(2;12) = 5.14; *P* = 0.024]. **b** For SUS1, there is a significant effect of genotype [ANOVA, *F* (2;12) = 18.39; *P* = 0.0002], treatment [ANOVA, *F* (1;12) = 17.10; *P* = 0.0014], yet there is no significant effect of the interaction of genotype: treatment [ANOVA, *F *(2;12) = 2.73; *P* = 0.11]. Protein lysates separated during SDS-PAGE electrophoresis were obtained by pooling the leaf material from at least 6 plants for each treatment from three independent experiments (*n* = 3). Bars represent the means ± SD, asterisks indicating a significant effect of chilling (Tukey’s HSD test): ***P ≤ 0.001

Western-blot analysis revealed no significant changes in the SPS relative expression between control (non-chilled) and chilled plants of Zm-T maize line (Fig. 1a), while in chilled plants of MG and Zm-S maize line significant changes in the SPS abundance compared to the control plants was noted (*P* < 0.001)—about 1.5-fold and twofold higher, respectively. In the case of SUS1, ANOVA showed no significant effect of the interaction for genotype: treatment [*F *(2;12) = 2.73; *P* = 0.11] (Fig. 1b).

### Immunogold localization of SPS and SUS

**Fig. 2:**
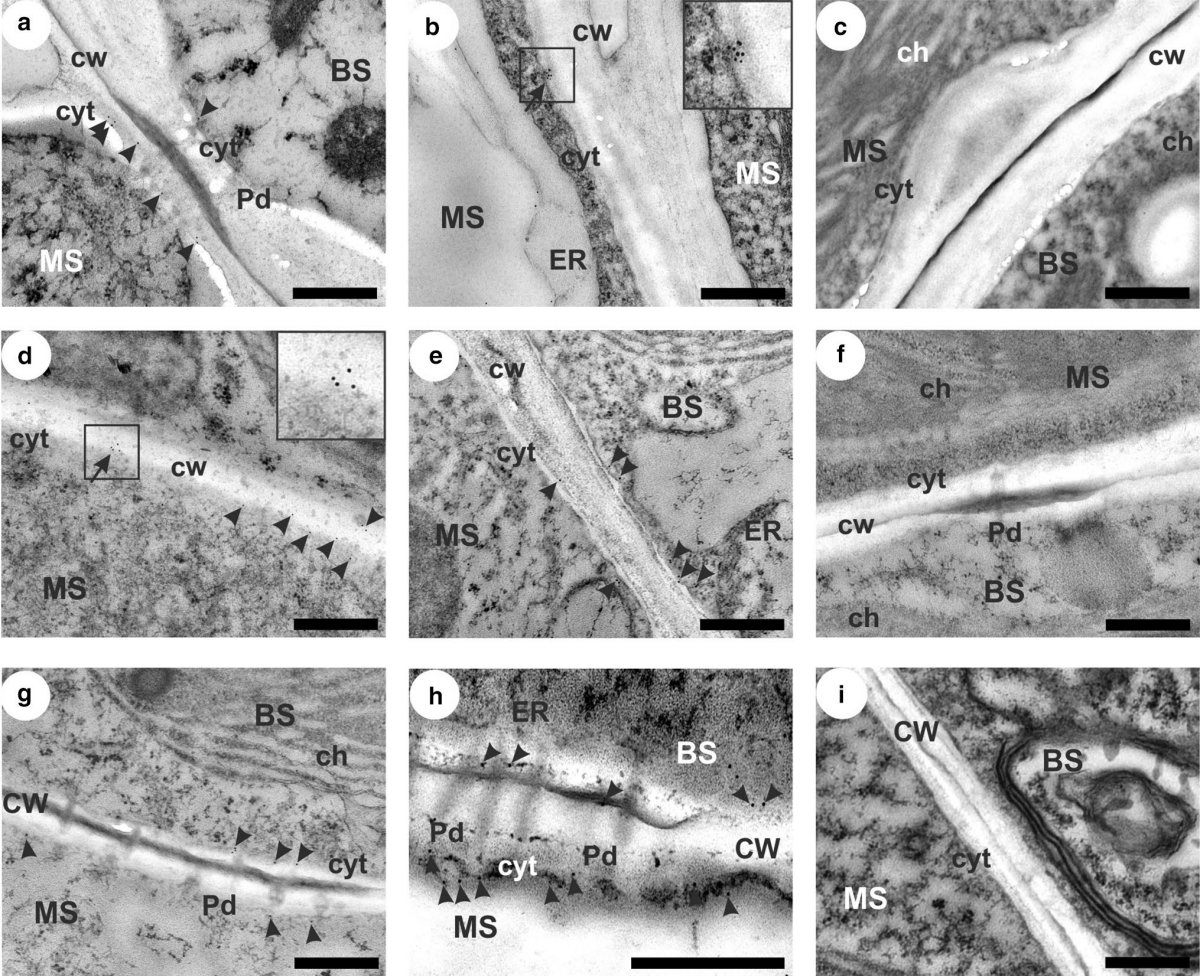
Examples of localization of sucrose phosphate synthase (SPS) in the leaves of *Miscanthus* × *giganteus*, MG, chilling-tolerant, Zm-T and chilling-sensitive maize line, Zm-S. Control (not-chilled plants) of MG (**a**), Zm-T line (**d**) and Zm-S maize line (**g**). Chilled-treated plants of MG (**b**), Zm-T line (**e)** and Zm-S maize line (**h**). Single gold particles (arrowheads) and the clusters of colloidal gold grains (arrows, more than three particles) visualizing anti-SPS antibody were mainly observed in the cytoplasm of mesophyll and bundle sheath cells, including areas near plasmodesmata (**a**, **g**, **h**). Note: more intense labelling in the cytoplasm of mesophyll cells of chilled plants of Zm-S maize line (**h**) compared to the control one (**g**). No signal was detected in the negative control, where the incubation with primary antibodies was omitted, in *Miscanthus* (**c**) and maize leaves (**f**, **i**). MS, mesophyll; BS, bundle sheath; VP, vascular parenchyma; ch, chloroplast; CW, cell wall; cyt, cytoplasm; ER, endoplasmic reticulum; Pd, plasmodesmata. Scale bar = 500 nm

In general, SPS visualized by gold particles was localized in the cytoplasm of mesophyll and bundle sheath cells of the leaves of both tested plant species (Fig. 2). In rare cases, single gold grains were also observed in other subcellular localizations (e.g., endoplasmic reticulum) and in other types of cells (e.g., vascular parenchyma), however, the signal intensity was very low, at the background level (data not shown). In MG plants, SPS was predominantly found in the mesophyll cells where single gold particles as well as clusters of grains were observed (Fig. 2a, b), whereas in both maize lines SPS epitopes were evenly distributed in the mesophyll and bundle sheath cells (Fig. 2d, e, g, h). Cold caused a marked increase in SPS labelling in the leaves of Zm-S maize line, particularly in the mesophyll cells (Fig. 2h). The negative control, where the incubation with primary antibodies was omitted, confirmed the correctness of the method used—no signal was observed in the leaves of MG (Fig. 2c) and both maize lines (Fig. 2f, i).

**Fig. 3:**
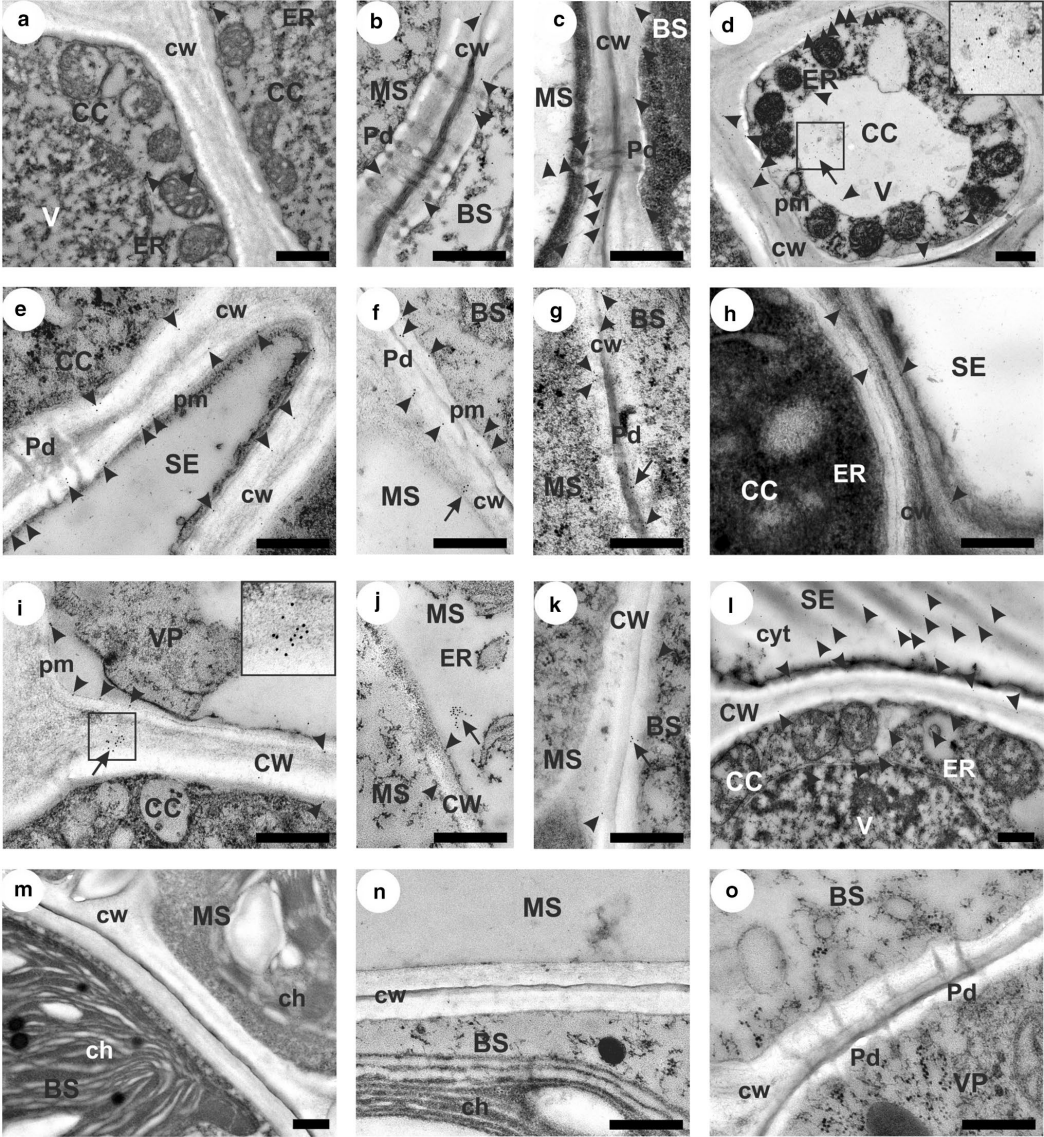
Localization of synthase sucrose 1 (SUS1) in the leaves of *Miscanthus* × *giganteus*, MG, chilling-tolerant, Zm-T and chilling-sensitive maize line, Zm-S. Control (not-chilled plants) of MG (**a**, **b**), Zm-T line (**e**, **f**) and Zm-S maize line (**i**, **j**). Chilled-treated plants of MG (**c**, **d**), Zm-T line (**g**, **h**) and Zm-S maize line (**k**, **l**). Single gold particles (arrowheads) and the clusters of colloidal gold grains (arrows, more than three particles) visualizing anti-SUS1 antibody were mainly observed along the plasma membrane in all types of cells tested (**a**-**l**), in the secondary cell wall (**b**-**g**, **i**, **k**), including areas near plasmodesmata (**b**, **c**, **f**, **g**), at endoplasmic reticulum (**a**, **d**, **j**), as well as in the vacuole (**d**) and cytoplasm (**l**). Note: more intense labelling in chilled plants of MG compared to the control one, in the secondary cell wall of mesophyll cells (**b** *vs.* **c**) and vacuole of companion cells (**a** *vs*. **d**). Negative control for *Miscanthus* (**m**) and maize leaves (**n**, **o**). MS, mesophyll; BS, bundle sheath; VP, vascular parenchyma; CC companion cell; SE, sieve element; ch, chloroplast; CW, cell wall; cyt, cytoplasm; ER, endoplasmic reticulum; pm, plasma membrane; Pd, plasmodesmata; V, vacuole. Scale bar = 500 nm

SUS1 was localized in different cell types and in various organelles in the leaves of both tested plant species (Fig. 3). This protein was mainly found along the plasma membrane in all types of cells tested (Fig. 3a–l), in the secondary wall (Fig. 3b–g, i, k), as well as in the cell wall areas near plasmodesmata (Fig. 3b, c, f, g), also at the endoplasmic reticulum (Fig. 3a, d, j), in the vacuole (Fig. 3d) and in the cytoplasm (Fig. 3l). In MG and Zm-T plants, SUS1 was more frequently observed in the cell wall, especially at mesophyll/bundle sheath interface, including plasmodesmata areas – so-called ‘pit fields’ (Fig. 3b, c, f, g). In the case of MG plants, SUS1 was also found in the vacuoles of companion cells (Fig. 3d), while in the Zm-S maize line SUS1 labelling was more intense in the cell wall of companion cells (Fig. 3i), and at endoplasmic reticulum, particularly in the mesophyll cells (Fig. 3j), as well as in the cytoplasm of sieve elements (Fig. 3l). Cold led to an increase in the SUS1 labelling in MG leaves in the secondary wall between mesophyll and bundle sheath (Fig. 3b *vs*. c) and in the vacuoles of companion cells (Fig. 3a *vs*. d). In both maize lines, such differences in the intensity of SUS1 labelling were not observed. Simultaneously, the negative control, where the incubation with primary antibodies was omitted, showed no labelling in the cellular components of the MG leaves (Fig. 3m) and both maize lines (Fig. 3n, o).

### Sucrose content

**Fig. 4:**
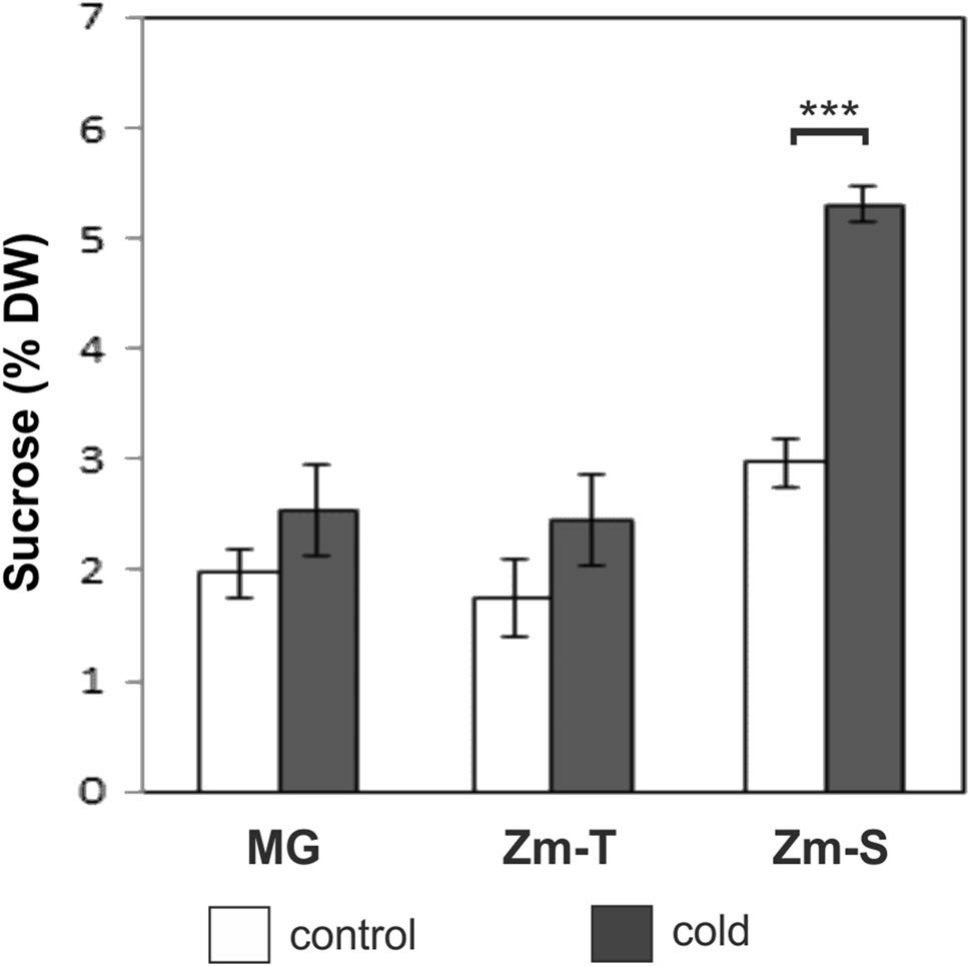
The level of sucrose analyzed by GC in the leaves of the control (white bars) and chilled (grey bars) plants of *Miscanthus* × *giganteus* (MG), chilling-tolerant (Zm-T) and chilling-sensitive maize line (Zm-S). The sucrose content is expressed as % leaf dry weight (DW). The significant effect of genotype [ANOVA, *F *(2;12) = 79.03; *P* < 0.0001], treatment [ANOVA, *F *(1;12) = 66.89; *P* < 0.0001] and effect of the interaction of genotype: treatment were noted [ANOVA, *F *(2;30) = 14.76; *P* < 0.0001]. Results represent six independent extractions (*n* = 6) with 80% (v/v) ethanol. Bars represent the means ± SD, asterisks indicating a significant effect of chilling (Tukey’s HSD test): ***P ≤ 0.001

Gas chromatography analysis revealed no significant changes in the sucrose content in the leaves of control and chilled plants of MG and Zm-T maize line (Fig. 4), whereas cold caused a significant increase by 78% in the sucrose amount in the Zm-S maize line treated with low temperature (*P* < 0.001).

### FTIR spectroscopy

**Fig. 5:**
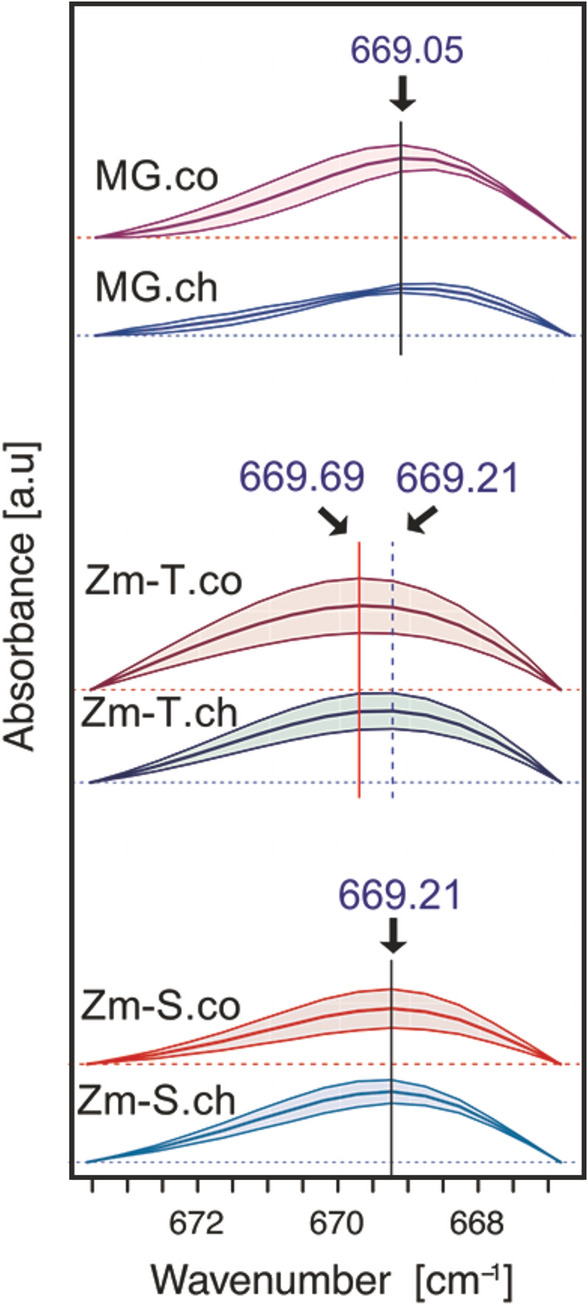
Absorbance of infrared spectra (means ± 95% confidence intervals) of cell wall isolated from the 3^rd^ leaves in the control (red/.co) and chilled (blue/.ch) plants of *Miscanthus* × *giganteus* (MG), chilling-tolerant (Zm-T) and chilling-sensitive maize line (Zm-S). The spectra are given for the region between c. 667 and 673.5 cm^−1^ wavelength assigned to the OH out-of-plane bending vibrations in cellulose. The significant genotype effect for the peak position [ANOVA, (*F *(2;29) = 13.571; *P* < 0.01)], and the significant genotype: treatment interaction (*F *(2;29) = 4.009; *P* = 0.029) were noted. The confidence intervals from Tukey.HSD post-hoc test for estimated difference of means between the maximum peak position of chilled and control plants were as follows: MG [95% CIs (− 0.2739, 0.3772), *P* = 0.99], Zm-T [95% CIs (− 0.0055, 0.6455), *P* = 0.056] and Zm-S [95% CIs (− 0.2295, 0.4534), *P* = 0.91]. Positions of mean maximum absorbances are marked with arrows. The spectra are shifted in absorbance for clarity

Fourier transform infrared spectroscopy (FTIR) analysis, a technique sensitive both to biochemical profile (functional groups presence) and the chemical structure of the components, affected by non-covalent interactions was used to detect the potential modifications in the cellulose chain arrangement associated with the response to cold. Cellulose assigned infrared bands involve several frequency regions between the 600 cm^−1^ and 4000 cm^−1^ (Nelson and O’Connor 1964) among which one band specifically attributed to out-of-plane O–H bending vibrations in cellulose is around 670 cm^−1^ (Kondo and Sawatari 1996). The spectra of both examined species for control and chilled plants trimmed to the region between 667 and 673.5 cm^−1^ are given in Fig. 5. The chilling caused a slight shift (*P* = 0.056) of the maximum absorbance from 669.69 cm^−1^ towards lower frequency (669.21 cm^−1^) in the plants of Zm-T maize line. No such frequency shifts were observed either, for MG (*P* = 0.99) or Zm-S line (*P* = 0.91).

### Starch grains

**Fig. 6:**
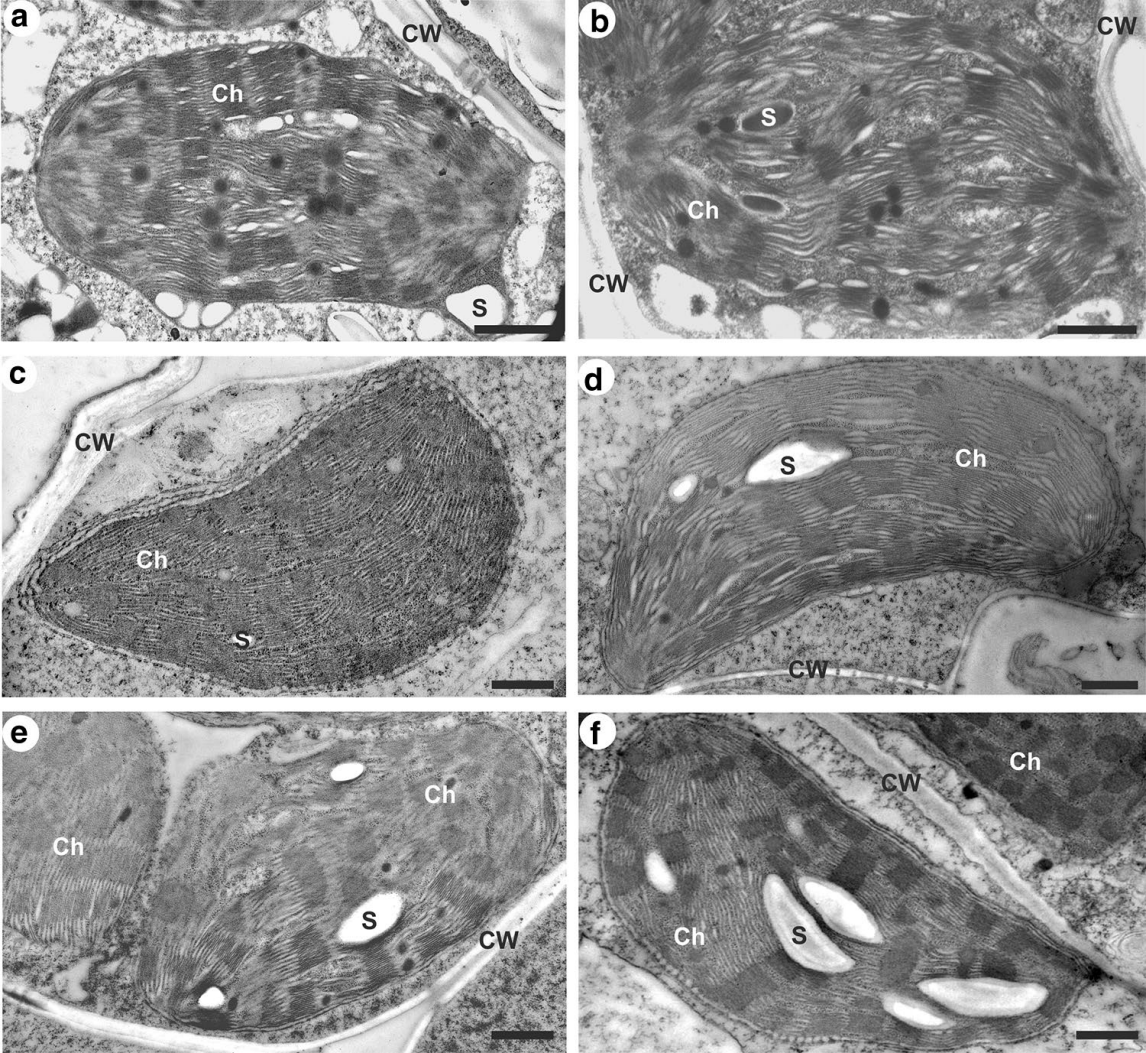
Ultrastructure of mesophyll chloroplasts with starch grains arrangement in the control (**a**, **c**, **e**) and chilled (**b**, **d**, **f**) plants of *Miscanthus* × *giganteus* (MG), chilling-tolerant (Zm-T) and chilling-sensitive maize line (Zm-S). In the chilled leaves of Zm-S line (**f**) more starch grains are observed than in the control plants of this maize line (**e**). Ch, chloroplast; CW, cell wall; S, starch grain. Scale bar = 1 µm

**Fig. 7:**
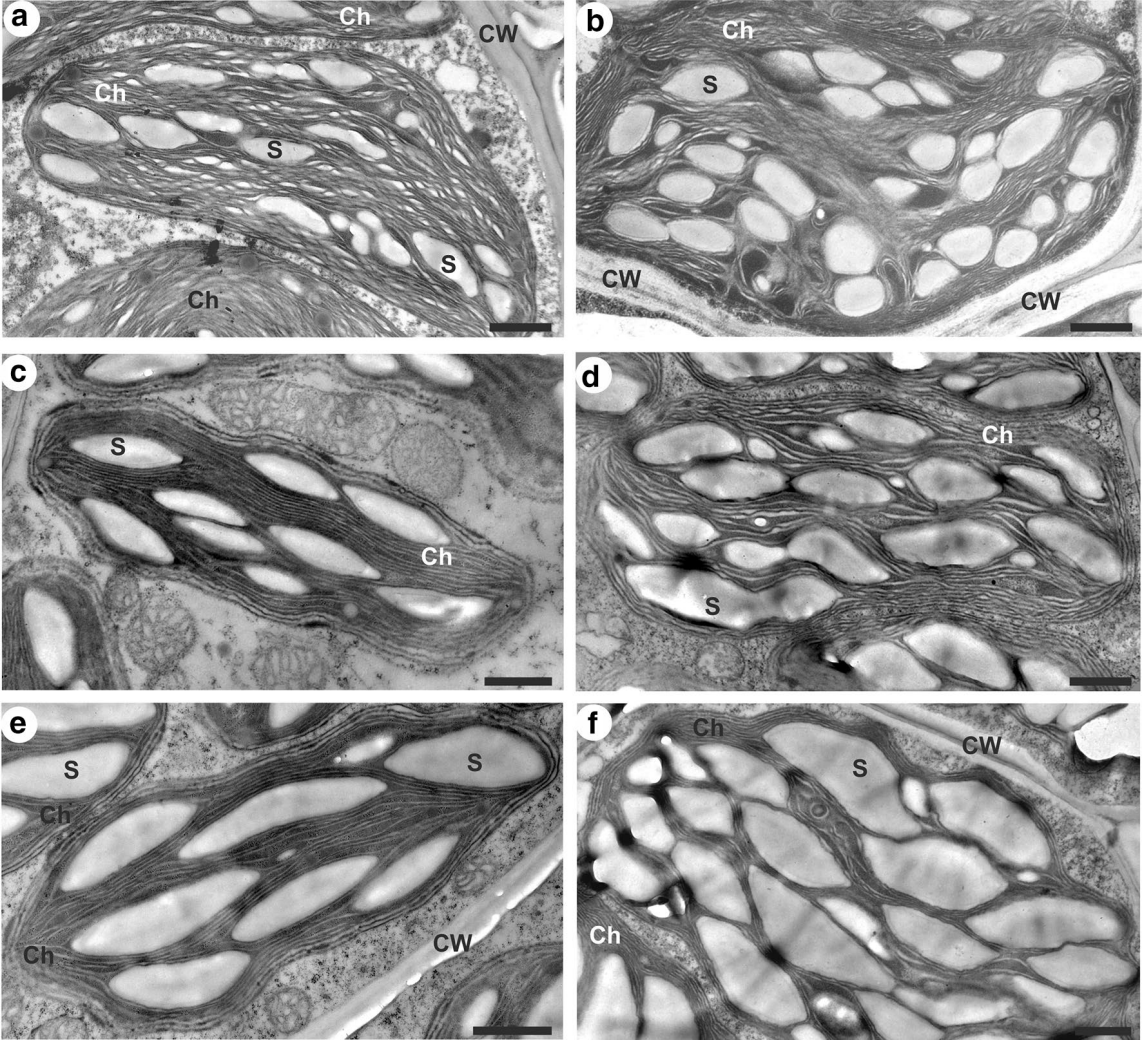
Ultrastructure of bundle sheath chloroplasts with starch grains arrangement in the control (**a**, **c**, **e**) and chilled (**b**, **d**, **f**) plants of *Miscanthus* × *giganteus* (MG), chilling-tolerant (Zm-T) and chilling-sensitive maize line (Zm-S). Note the extensive starch grains filling in the chloroplast of chilled plants of Zm-S maize line (**f**). Ch, chloroplast; CW, cell wall; S, starch grain. Scale bar = 1 µm

**Fig. 8:**
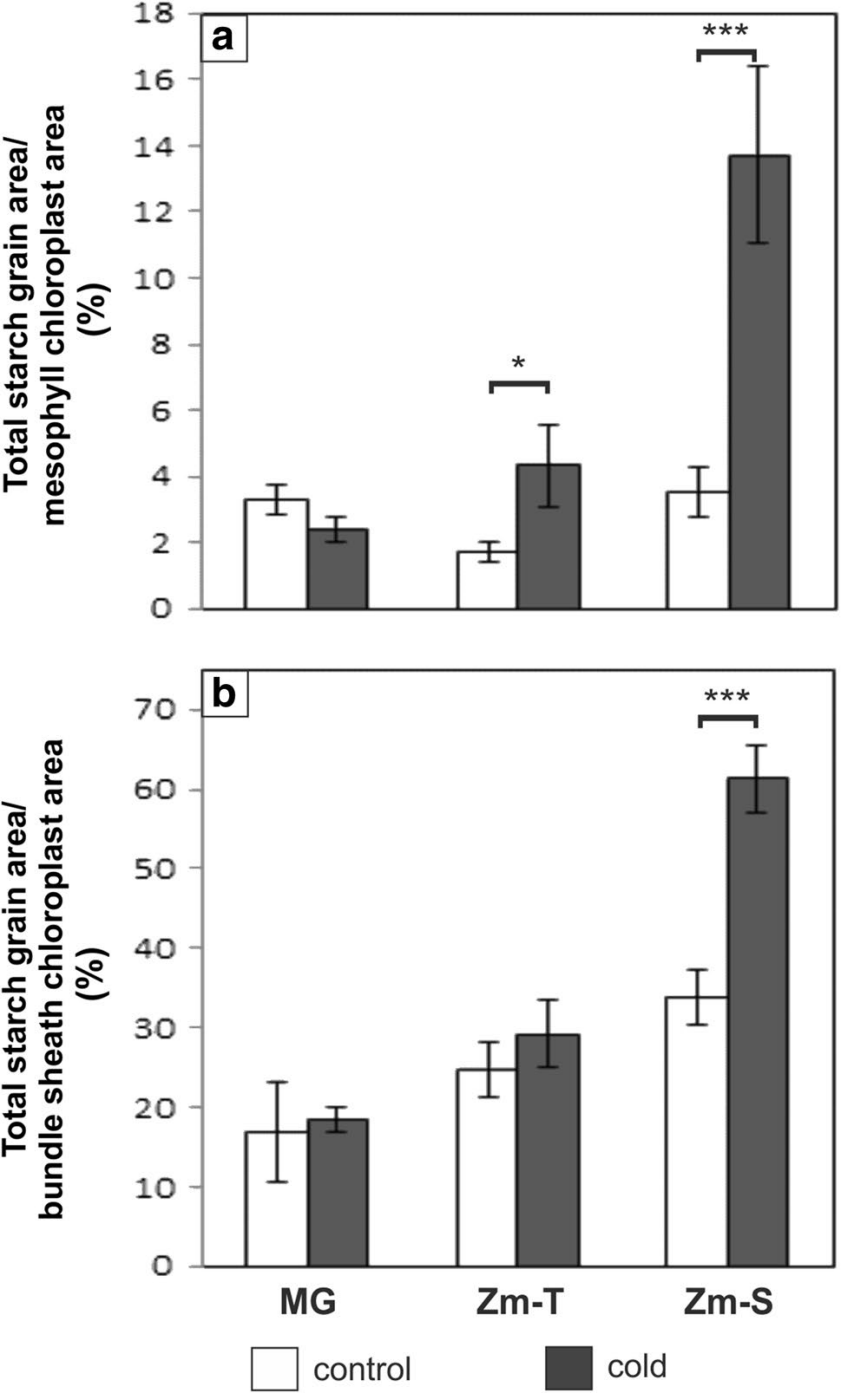
Total starch grain area in the chloroplasts of mesophyll (**a**) and bundle sheath cells (**b**) in the control (white bars) and chilled (grey bars) plants of *Miscanthus* × *giganteus* (MG), chilling-tolerant (Zm-T) and chilling-sensitive maize line (Zm-S). Values are expressed as % of total area of starch grain in the total area of chloroplast. **a** Significant effect of genotype [ANOVA, *F *(2;102) = 73.45; *P* < 0.0001], treatment [ANOVA, *F *(1;102) = 80.78; *P* < 0.0001] and of the interaction of genotype: treatment [ANOVA, *F *(2;102) = 54.89; *P* < 0.001]. **b** Significant effect of genotype [ANOVA, *F *(2;102) = 153.35; *P* < 0.0001], treatment [ANOVA, *F *(1;102) = 61.27; *P* < 0.0001] and of the interaction of genotype: treatment [ANOVA, *F *(2;102) = 31.41; *P* < 0.0001]. For each treatment, the total area of starch grain in the chloroplasts was measured from at least thirty Kranz mesophyll or bundle sheath cells from six plants with three independent experiments (*n* = 18). Bars represent the means ± SD; asterisks indicating a significant effect of chilling (Tukey’s HSD test): *P ≤ 0.05; ***P ≤ 0.001

The electron micrographs of the chloroplasts (Figs. 6, 7) were consistent with the general C_4_ plants anatomy (NADP-ME subtype) with a centrifugal arrangement of bundle sheath chloroplasts (von Caemmerer and Furbank 2003). In the mesophyll and bundle sheath chloroplasts of both tested plant species, starch grains were of different sizes and had various shapes, usually close to the oval (Figs. 6, 7). In the case of chilled plants of Zm-S maize line, more starch grains were noted in the mesophyll chloroplasts compared to the control plants (Fig. 6f). Also, in the chloroplasts of bundle sheath cells a clear increase in the amount of starch grains was noted in this maize line under cold condition (Fig. 7f). The significant increase in total starch grain area of chloroplasts in both cell types was demonstrated in the chilled plants of Zm-S maize line (*P* < 0.001). An about fourfold and 1.8-fold higher amount of starch granules was noted for mesophyll and bundle sheath chloroplast, respectively (Fig. 8). In turn, in chilled plants of Zm-T maize line the increase in total starch grains area was observed only in the chloroplasts of mesophyll cells (*P* = 0.018). The cold did not cause changes in the amount of starch grains in the chloroplasts of both cell types in MG plants.

## Discussion

Sucrose phosphate synthase (SPS) is assumed to be a key regulator for controlling biosynthesis of sucrose in the source tissues; sucrose is then transported to the sink organs for the utilization, storage or as an element of the signalling pathway (Winter and Huber 2000). The accumulation of soluble sugars, including sucrose, is known as one of the mechanisms of plant adaptation to dehydration and freezing stress. Under these conditions, sugars as osmoprotectants may regulate the osmotic potential of cells, and can also stabilize membranes, through interaction with the lipid layer (reviewed by Sami et al. 2016).

In our work, the higher abundance of SPS (Fig. 1a) with more intense labelling in the cytoplasm of mesophyll cells (Fig. 2h) in the leaves of the chilled Zm-S line reflected the increase in sucrose content in leaves of this maize line (Fig. 4). Similarly, in our previous studies, in another chilling-sensitive maize line the accumulation of sucrose under cold conditions has been noted (Bilska-Kos et al. 2017) which was probably caused by the inhibition of transport processes via closure of plasmodesmata in the photosynthetic pathway (Bilska and Sowiński 2010). It has been previously found that the carbon transfer between the C_3_ and C_4_ cycle in C_4_ plants is slower at low temperature (Long 1983), and the transport of sucrose and other low-molecular compounds in the ER cylinder of plasmodesmata is less efficient (Gamalei et al. 1994). In this scenario, the sucrose which was unable to pass through blocked plasmodesmata to the phloem loading site, i.e. to the complex of companion cells/sieve elements, was accumulated in the Kranz mesophyll, bundle sheath and vascular parenchyma cells where lower osmotic potential was observed (Bilska-Kos et al. 2017). This reaction occured in a relatively short time—just after 1 h of chilling, the closure of plasmodesmata was observed (Bilska and Sowiński 2010). Hence, it can be assumed that the rapid accumulation of sucrose observed in chilled plants of Zm-S maize line (Fig. 4) is a direct response to cold stress associated with the blocking of intercellular transport rather than the element of the adaptation to low-temperature conditions. In contrast, the increase in sucrose content under prolonged chilling (7 days) in both, chilling-tolerant and chilling-sensitive maize lines, can be considered as a manifestation of the ability to adapt to moderate cold conditions of both maize lines (Sowiński et al. 1999). It should be noted, that in some cases the overaccumulation of sucrose is not accompanied by a high abundance of SPS, and conversely. For instance, the increase in the sucrose content in maize leaves was not a result of the changes in SPS activity (Lunn and Hatch 1997). Similarly, knockout mutants of rice (*OsSPS1*) with reduced SPS activity showed no changes in the content of sugars, including starch/sucrose ratio and in the overall plant growth (Hashida et al. 2016). In addition, mRNA levels of two SPS forms in rice were negatively correlated with sucrose content (Okamura et al. 2011). However, the higher SPS protein expression in cold-acclimated barley plants was associated with freezing tolerance in tested genotypes (Rapacz et al. 2008). Hence, it may be possible that under certain conditions, a higher SPS protein level provides sufficient sucrose synthesis, even at very low enzyme activity.

On the other hand, the part of the sucrose pool synthetized by SPS may be used for the production of the cellulose and starch by SUS (Im 2004). This may partly explain the high SPS level in chilled MG plants compared to the control ones (Fig. 1a) with simultaneously observed unchanged level of sucrose in this plant species (Fig. 4). It is also possible, that another gene family may regulate SPS activity in *Miscanthus* than in maize because such diversity in gene expression pattern has been demonstrated for several species within grasses (Castleden et al. 2004).

The second enzyme tested in this work, sucrose synthase (SUS), is most often considered as an enzyme challenging UDP-glucose to cellulose synthesis, particularly in the stage of intensive secondary cell wall formation (reviewed by Haigler et al. 2001). In our previous work, cold led to the reduction of the crystalline cellulose content but, at the same time, caused the increase in the cell wall thickness of bundle sheath cells in the MG plants (Bilska-Kos et al. 2018). In the present work, the increase in SUS1 labelling in the secondary cell wall between mesophyll and bundle sheath cells (Fig. 3c) may be evidence for more intensified activity of this enzyme in these local (micro) areas. Similarly, higher activity of SUS enzymes in hybrid poplar plants resulted in a thicker cell wall of xylem and greater crystallinity of this wall type, which consequently led to increased wood density (Coleman et al. 2009). It is also possible that SUS1 has a predominant role during increased energy demand under abiotic stress in *Miscanthus* plants, due to the fact, that different genes encoding various SUS isoforms may have specialized function depending on the plant species, developmental stage or external factors (Wang et al. 2015). On the other hand, it should be realized that the increase in SUS1 abundance, which was ‘captured’ on the microscopic images may have temporal character during a dynamic carbon partitioning process.

The FTIR spectroscopy quantitative analysis showed a slight shift towards lower values at about 670 cm^−1^, attributed to cellulose (Kondo and Sawatari 1996; Abidi et al. 2014), exclusively for the Zm-T maize line in the response to low temperature (Fig. 5). This indicates an increase in the O–H⋅⋅⋅O hydrogen bonding between cellulose chains (Kondo and Sawatari 1996; Ilharco et al. 1997) that may reflect the onset of formation of slightly more compact and/or more crystalline structure in Zm-T maize line.

In turn, the relatively high intensity of SUS1 labelling in the phloem cells of the Zm-S line (Fig. 3l) may be the result of the phloem loading inhibition observed in the other chilling-sensitive maize material (Bilska and Sowiński 2010), and may not be relevant for the synthesis of cell wall components. Such cellular localization of SUS may indicate the predominant role of this enzyme in the loading and unloading of sucrose in the phloem, which has been previously demonstrated in rice seeds (Wang et al. 1999), as well as in maize and citrus leaves (Nolte and Koch 1993).

In addition to the role in the cellulose synthesis, SUS participates in the supply of substrates such as ADP-glucose, for the starch synthesis. Under cold stress, the increase in starch content is mainly observed, however, this response may depend on other factors, i.a. experimental conditions (reviewed by Thalmann and Santelia 2017). This mechanism may be associated with e.g., cold-induced changes in the intra-/inter-cellular transport, in the phloem loading and in the long-distance transport on the path: source (leaves)—sink (roots), as well as the modification of the osmotic/water potential of cells (Gamalei et al. 1994; Čiamporová and Trgiňová 1996; Bilska and Sowiński 2010; Bilska-Kos et al. 2016b, 2017). The starch is primarily accumulated in chloroplasts and the consequence of both, direct and indirect (including changes in the starch grains organization) cold effects, on the structural modifications of chloroplasts have been widely reported (Čiamporová and Trgiňová 1996; Kutík et al. 2004; Skupień et al. 2017). Generally, the cold-induced starch accumulation in the chloroplasts is accompanied by the changes in the arrangement of thylakoids, in the amount of plastoglobuli and in granal organization (Čiamporová and Trgiňová 1996; Kutík et al. 2004). In our case, the marked increase in total starch grain area was observed in the Zm-S maize line, in both mesophyll and bundle sheath chloroplasts (Figs. 6, 7, 8). Thus, it may be supposed that the changes in the structure of chloroplasts leading to disorders in the photosynthetic apparatus can affect photosynthesis and the transport of photosynthetic products, as has been previously observed for other chilling-sensitive maize line (Bilska and Sowiński 2010; Bilska 2013; Bilska-Kos et al. 2016a). The effect of cold on the photosynthetic apparatus was also visible in the 5th maize leaf—the starch grains were noted in bundle sheath chloroplasts in the leaves of the chilling-sensitive line (Sowiński et al. 2005), what has been confirmed by gas–liquid chromatography in another work (Sowiński et al. 1999). These results were explained by deepening inorganic phosphorus deficiency in this maize line under cold conditions. In turn, in the other work, starch grains completely disappeared after 7 days of cold treatment at 6 °C in the chloroplast of bundle sheath cells in the leaves of chilling-sensitive maize line (Penjalinan) while in chilling-tolerant line (Z7) number of starch grains was increased (Čiamporová and Trgiňová 1996). Among different maize lines perhaps a variation in the metabolism of starch under cold stress conditions might occur, highly specific to each genotype. On the other hand, the accumulation of starch in the chloroplasts, as an energy storage material, may be a manifestation of the ability to adapt to the stress conditions. For instance, the up-regulated genes related to starch metabolism could protect the young shoots of tea (*Camellia sinensis*) against cold-induced damages and participate in the general process of the acclimatization to low temperature (Hao et al. 2018). In turn, in *Arabidopsis* the cold-enhanced breakdown of starch was associated with freezing tolerance manifested by starch-depended maltose accumulation which can protect the photosynthetic electron transport chain during stress (Kaplan and Guy 2005). Thus, it is possible that the observed in this study slight increase in the total starch grain area in the mesophyll chloroplasts of Zm-T maize line (Fig. 8) had no significant effect on these organelles structure (Fig. 6d), and may indicate the activation of certain defensive paths of the adaptive mechanism to cold stress in this maize line.

## Conclusion

This work demonstrated that, in both the most chilling-tolerant (MG) and the least chilling-tolerant genotype (Zm-S maize line), SPS content was increased at low temperature, but this was most likely associated with different pathways of cold acclimation. In the case of Zm-S line, the higher abundance of SPS resulting in the increase in sucrose level, as well as the increase in total starch grain area in the chloroplasts, may indicate the changes in the general sugar metabolism under cold conditions. These changes may be associated with the photosynthesis and transport process inhibition, as well as osmotic potential modifications (Bilska and Sowiński 2010; Bilska-Kos et al. 2017). Furthermore, the higher abundance of SPS in the chilled leaves of MG plants could lead to increased supply of sucrose and its immediate use by SUS for the synthesis of cell wall material. This may be related to local remodeling of cellulose as evidenced by the increase in the SUS1 labelling intensity in the cell wall between mesophyll and bundle sheath cells of chilled MG plants. Further research, including integrated analyses at physiological, biochemical and molecular levels of other enzymes involved in the sugar metabolism, can bring new elements to the ‘puzzle’ that forms the overall cold-response of C_4_ plants.

## *Author contribution statement*

AB-K defined the research problem, designed the experiments and wrote the manuscript. JMy performed Western-blot experiments and the analysis of their results. AB-K prepared the material for electron microscopy, carried out immunogold experiments and microscopic observations. SS performed a quantitative analysis of starch grains. JMa prepared samples for gas-chromatography analysis and carried out gas-chromatography experiments. PO performed gas-chromatography analysis. AB-K and JZ performed FTIR measurements. JZ analyzed FTIR spectra and wrote FTIR section. All authors read and approved the final version of the manuscript.

## Abbreviations

FTIR: Fourier transform infrared spectroscopy
MG: *Miscanthus* × *giganteus*
SPS: Sucrose phosphatate synthase
SUS1: Sucrose synthase 1
Zm-S: Chilling-sensitive maize line
Zm-T: Chilling-tolerant maize line
